# *ANKRD26* Gene Mutation and Thrombocytopenia—Is the Risk of Malignancy Dependent on the Mutation Variant?

**DOI:** 10.3390/hematolrep17040037

**Published:** 2025-07-24

**Authors:** Eirik B. Tjønnfjord, Kristian Tveten, Signe Spetalen, Geir E. Tjønnfjord

**Affiliations:** 1 Department of Internal Medicine, Østfold Hospital Trust, 1714 Grålum, Norway; 2 Department of Haematology, Oslo University Hospital, 0372 Oslo, Norway; gtjonfj@ous-hf.no; 3 Department of Medical Genetics, Telemark Hospital Trust, 3710 Skien, Norway; ktveten@sthf.no; 4 Department of Pathology, Oslo University Hospital, 0372 Oslo, Norway; signe.spetalen@ous-hf.no; 5 Institute of Clinical Medicine, University of Oslo, 0372 Oslo, Norway; 6 Section of Cancer Cytogenetics, Institute of Cancer Genetics and Informatics, Oslo University Hospital, 0372 Oslo, Norway

**Keywords:** thrombocytopenia, inherited, mutations, genes, ANKRD26

## Abstract

*Background and Clinical Significance:* Inherited thrombocytopenia (IT) is a heterogeneous group of disorders caused by mutations in over 45 genes. Among these, ANKRD26-related thrombocytopenia (ANKRD26-RT) accounts for a notable subset and is associated with variable bleeding tendencies and an increased risk of myeloid malignancies. However, the extent of this oncogenic risk appears to vary between specific gene variants. Understanding the genotype–phenotype relationship is essential for patient counseling and management. This report presents a multigenerational family carrying the rare c.−118C > G variant in the 5′ untranslated region of ANKRD26, contributing to the discussion on variant-specific cancer predisposition. *Case Presentation:* Two sisters aged 57 and 60 presented with lifelong bleeding diathesis and moderate thrombocytopenia. Their symptoms included easy bruising, menorrhagia, and excessive postoperative bleeding. Genetic testing confirmed heterozygosity for the ANKRD26 c.−118C > G variant. Bone marrow analysis revealed abnormal megakaryopoiesis without evidence of dysplasia or somatic mutations. One sister underwent major surgery without complications when managed with prophylactic hemostatic therapy. Their family history included multiple female relatives with similar symptoms, although formal testing was limited. Notably, none of the affected individuals developed hematologic malignancy, and only one developed esophageal cancer, with no current evidence linking this variant to solid tumors. *Conclusions:* This case underscores the importance of distinguishing between ANKRD26 variants when assessing malignancy risk. While ANKRD26-RT is associated with myeloid neoplasms, the c.−118C > G variant may confer a lower oncogenic potential. Variant-specific risk stratification and genetic counseling are crucial for optimizing surveillance and avoiding unnecessary interventions in low-risk individuals.

## 1. Introduction

Inherited thrombocytopenia (IT) is part of a growing group of disorders caused by molecular defects involving more than 45 different genes [1,2,3]. The prevalence of IT is estimated at 2–3 per 100,000 individuals, although this is likely an underestimate. Milder cases often go unrecognized, and more severe cases may be misdiagnosed as acquired thrombocytopenia [4].

Inherited thrombocytopenias often present diagnostic challenges due to their clinical overlap with acquired forms, such as immune thrombocytopenia (ITP), myelodysplastic syndromes, or drug-induced thrombocytopenia. In the absence of syndromic features or a family history, these inherited forms may remain undetected for years. Misdiagnosis may lead to inappropriate treatment, including corticosteroids, immunoglobulins, or even splenectomy. Consequently, greater awareness and access to genetic testing are essential for accurate diagnosis and management of IT.

ANKRD26 (Ankyrin Repeat Domain-Containing 26)-related thrombocytopenia (ANKRD26-RT) accounted for 10% of IT cases in a large Italian cohort, making it one of the most common inherited thrombocytopenias [5]. However, the true prevalence for ANKRD26-RT remains unknown. All reported individuals with ANKRD26-RT have an affected parent, but there is significant phenotypic variability within families, which is consistent with variable expressivity. More than 70 families, encompassing over 200 affected individuals, have been described in the literature [4].

ANKRD26-RT is classified as a non-syndromic IT with a predisposition to myeloid neoplasms [6]. The majority of the causative variants are located in a 19-nucleotide region of the 5′ untranslated region (5′UTR; c.-116 to c.−134), which serves as a critical binding site for the transcription factors RUNX1 (runt-related transcription factor 1) and FLI1 (friend leukemia integration 1, also known as transcription factor ERGB), which are essential regulators of thrombopoiesis. The lifetime risk of myeloid transformation remains uncertain, although an 8% risk has been estimated [7]. Importantly, not all sequence variants within ANKRD26-RT have been associated with malignant transformation [8]. It is also worth noting that not only single-nucleotide substitutions have been reported, but also larger truncated mutations in the N-terminal [9,10].

We report a multigenerational family affected by ANKRD26-RT due to the heterozygous c.−118C > G variant. At least four individuals were affected, and none developed a myeloid malignancy. This variant has only been described in two individuals previously: a 32-year-old woman [11] and one other person of unspecified age and gender [12]. In the only previously detailed case of the c.−118C > G variant, the affected woman exhibited moderate thrombocytopenia and mild bleeding symptoms, with no signs of hematologic malignancy during follow-up. These findings are consistent with our current report and suggest that this variant may carry a lower oncogenic potential compared to more frequently reported variants such as c.−128G > A or c.−127C > T. However, due to the rarity of this variant, definitive conclusions cannot be drawn yet [13].

## 2. Materials/Case

**Figure 1:**
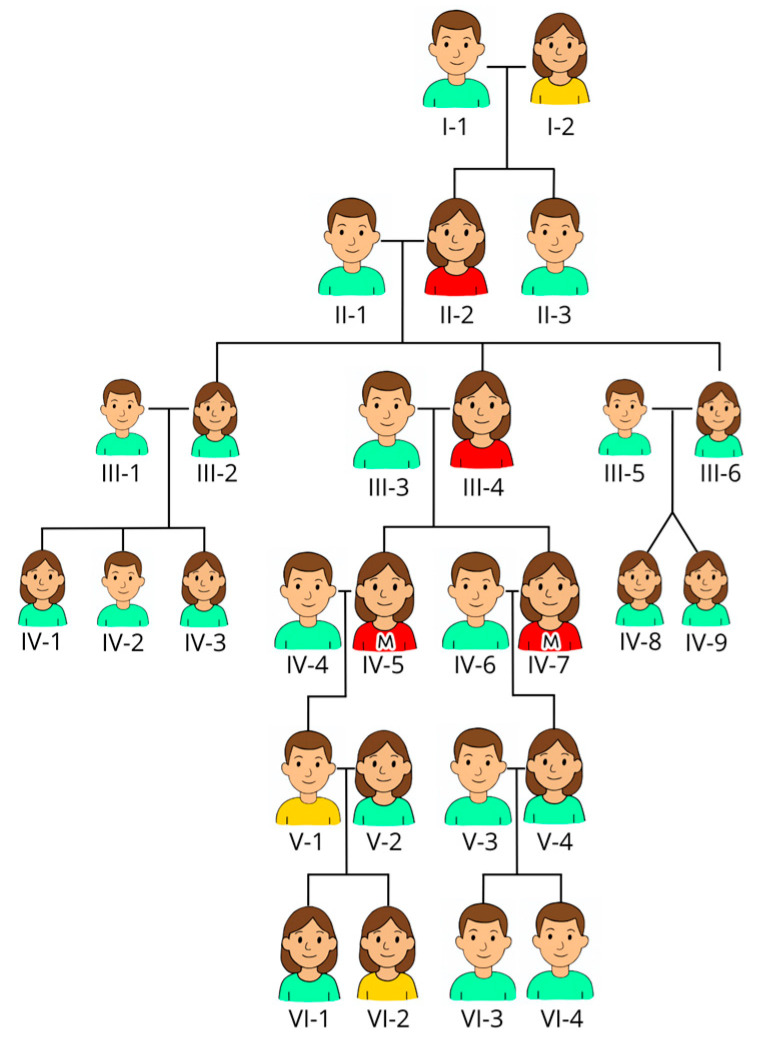
Pedigree of the family. The males have short hair, and the females have long hair. We have used the color of their shirts as an indicator of affections: red indicates confirmed thrombocytopenia (but mutation is only confirmed in the two sisters marked with M on their shirts, also known as probands); yellow indicates suspicion of thrombocytopenia (likely but not confirmed); and green indicates unaffected persons. *Proband I (IV-5. Proband II (IV-7)*.

Two sisters, aged 57 and 60, were referred for an evaluation of chronic thrombocytopenia and lifelong bleeding tendencies. Laboratory investigations confirmed moderate thrombocytopenia (see Table 1). Both reported easy bruising from early childhood, and both experienced menorrhagia and iron-deficiency anemia after menarche. Proband I (IV-5) had prolonged bleeding following dental extraction and suffered major hemorrhage after cesarean section; she was advised against further pregnancies. Proband II (IV-7) underwent hysterectomy at age 38 due to menorrhagia and experienced severe postoperative bleeding (see Figure 1 for family tree).

Their maternal history revealed similar symptoms. Their mother (III-4) and grandmother (II-2) had documented thrombocytopenia (but the platelet count is not available), easy bruising, and menorrhagia. Their great-grandmother (I-2) died from postpartum hemorrhage, but detailed blood counts were not available at that time, while their great-grandfather (I-1) lived to an advanced age with no signs of bleeding disorder. V-1 has bleeding diathesis along with his daughter (VI-2), but he has refused assessment and has not been tested in terms of either blood counts or mutation status.

Although IT was suspected clinically, the diagnosis was confirmed in 2020 when molecular analysis identified the c.−118C > G variant in ANKRD26 (NM_014915.3).

After IT was suspected, IV-5 underwent major dental surgery and IV-7 received a hip arthroplasty without bleeding complications when treated prophylactically with desmopressin, tranexamic acid, and platelet transfusion. IV-7 died at age 74 from esophageal cancer. Bone marrow analysis performed in 2020 revealed a normal female karyotype (46, XX) and no somatic mutations on the Illumina TruSight Myeloid Panel. The trephine biopsy was slightly hypercellular. The majority (50–60%) of the megakaryocytes were abnormal with two hypolobular nuclei (mono- and bilobular). CD34 + cells accounted for 3–4% of the bone marrow cellularity, and they had a normal phenotype as assessed by flow cytometry. IV-5 remains in good health at 78. Her son (V-1) and granddaughter (VI-2) experience mild bleeding symptoms but have declined further investigation.

## 3. Methods and Results/Conclusions

### 3.1. Methods

Whole-exome sequencing was performed using Twist Exome 2.0 (Twist Bioscience, San Francisco, CA, USA) and NextSeq500 (Illumina, San Diego, CA, USA). We applied a standard bioinformatic approach for base calling (RTA, Dubai, United Arab Emirates), read alignment (BWA), and variant detection (best practices variant calling using GATK).

The Illumina’s TruSight Myeloid Sequencing Panel (Illumina, San Diego, CA, USA) was used to identify somatic mutations in the bone marrow from proband IV-7. The panel covers 15 full genes (exons only) and 39 additional genes that are oncogenic hotspots. Alignment and variant calling were performed against GRCh37/hg19 with MiSeq Reporter Software v.2.6.3.2 (Illumina). SNVs and indels <60 bp were annotated by VariantStudio v.2.2. Pathogenicity was determined by using relevant databases. Sanger sequencing was not carried out.

### 3.2. Conclusions

Although patients with ANKRD26-RT generally have only mild bleeding symptoms, invasive procedures still pose a risk. There are no consensus guidelines for periprocedural hemostasis, but most case series recommend prophylactic measures such as desmopressin, antifibrinolytics, and platelet transfusion when platelet counts fall below 50 × 10^9^/L or in the setting of major surgery. The effective use of this regimen in IV-5 and IV-7 underscores the importance of individualized planning. Long-term management may include iron supplementation for chronic blood loss and gynecologic interventions for menorrhagia.

Thrombocytopenia in ANKRD26-RT is fully penetrant, but is typically mild to moderate with platelet counts ranging from 30 to 150 × 10^9^/L (mean ~48 × 10^9^/L). Counts below 10 × 10^9^/L are rare. Bleeding symptoms are often mild and mucocutaneous in nature, with easy bruising, epistaxis, and menorrhagia being common. Given the subtle phenotype and the reliance on molecular diagnostics, which may not be widely available, the diagnosis is often missed or delayed.

Although pathogenic variants in ANKRD26 have been associated with an increased risk of myeloid malignancies, this risk appears to vary by specific sequence variant. A case series of 118 individuals with confirmed or probable ANKRD26-RT reported an 8% incidence of myeloid malignancy. One report suggested a 24-fold increased risk [8,12], although this may reflect ascertainment bias. Notably, some variants, including c.−118C > G, have not been associated with malignancy to date.

While myeloid malignancies are the primary concern in ANKRD26-RT, there have been isolated reports of other cancers, such as solid tumors, in affected individuals [13]. However, the causal relationship between ANKRD26 variants and these malignancies remains elusive. In our case, IV-7 developed esophageal cancer at age 74, but there is currently no evidence linking the c.−118C > G variant with increased solid tumor risk. Larger cohort studies are needed to clarify whether any ANKRD26 variants predispose individuals to non-hematologic malignancies.

This family contributes to the growing body of evidence suggesting that the malignant potential of ANKRD26-RT may depend on the specific variant. Continued reporting of genotype–phenotype correlations is essential for refining prognostic estimates and guiding individualized management and surveillance strategies.

From a clinical perspective, identifying the specific ANKRD26 variant in affected families allows for more precise counseling regarding bleeding risk and cancer surveillance [14,15,16]. Cascade genetic testing can help identify at-risk relatives who may benefit from tailored monitoring or prophylaxis before surgical interventions. As variant-specific data accumulate, it may become possible to stratify patients by risk and refine surveillance protocols, potentially avoiding unnecessary anxiety or overtesting in low-risk individuals.

In summary, we present a multigenerational case of ANKRD26-RT caused by the c.−118C > G variant, with no cases of myeloid malignancy observed. Two other nucleotide changes have been reported in at least six other families (c.−118C > A and c.−188CT) [5,6,15,16,17]. Our findings support the hypothesis that not all ANKRD26 variants confer an equal risk of malignant transformation. This underscores the need for variant-specific risk stratification and the continued accumulation of genotype–phenotype data to improve patient care.

## Figures and Tables

**Table 1:** Blood cell counts at diagnosis and last follow-up of the probands.

	IV-5 14 January 2007	IV-5 21 November 2022	IV-7 08 January 2007	IV-7 01 December 2021	Reference Value
Hemoglobin g/dL	14.1	14.5	13.9	14.2	11.1–15.3
Erythrocytes 10^12^/L	4.6	4.9	4.4		3.9–5.2
MCV fL	88	89	94		82–98
Thrombocytes 10^9^/L	52	49	51	43	145–390
Leukocytes 10^9^/L	13.1	13.5	14.2	12.1	3.5–10.0
Granulocytes 10^9^/L	10.3	9.5	9.5	7.7	1.5–7.3
Lymphocytes 10^9^/L	2.2	2.8	3.1	2.8	1.1–3.3
Monocytes 10^9^/L	0.5	1.0	1.1	0.9	0.2–0.8

## Data Availability

All data regarding this publication can be found in the medical journal of the two probands, at Oslo University Hospital, Rikshospitalet.
